# Limbic Encephalitis: An Unusual Presentation on 18F-Fluorodeoxyglucose Positron Emission Tomography/Computed Tomography (18F-FDG PET/CT)

**DOI:** 10.7759/cureus.83988

**Published:** 2025-05-12

**Authors:** Grégory Omatuku Wetshosele, Salima Bouazza, Mario Manto, Massoud Moradi, Ivan Duran Derijckere

**Affiliations:** 1 Nuclear Medicine, University Hospital Center of Charleroi (CHU Charleroi), Charleroi, BEL; 2 Neurology, University Hospital Center of Charleroi (CHU Charleroi), Charleroi, BEL; 3 Radiology, University Hospital Center of Charleroi (CHU Charleroi), Charleroi, BEL

**Keywords:** anti-gaba-b-r, autoimmune limbic encephalitis, corticotherapy, inflammation activity, lung cancer, paraneoplasic syndrome, partial epilepsy

## Abstract

Limbic encephalitis is a relatively rare autoimmune neurological disorder, typically diagnosed based on clinical symptoms lasting less than three months, including seizures, memory deficits, psychiatric symptoms, bilateral mesial temporal lesions on MRI, inflammatory cerebrospinal fluid, and epileptiform activity on electroencephalogram (EEG). We report the case of a female patient who presented with an inaugural epileptic seizure, for which MRI, lumbar puncture, and cerebral positron emission tomography (PET) scan showed no pathological findings. The patient re-presented to the emergency department one month later with recurrent seizures. A subsequent PET scan revealed the emergence of a right mesial temporal lesion, and a hypermetabolic pulmonary lesion was identified, which was later diagnosed as small-cell lung carcinoma on histopathology. The patient showed favorable clinical improvement under Solu-Medrol treatment, and a follow-up imaging performed several months later showed complete resolution of the hypermetabolic cerebral lesion after chemotherapy. This case highlights an unprecedented early stage of limbic encephalitis, characterized by an initial absence of inflammation, suggesting that this might represent a nascent phase of the disease, which could be crucial for future management of similar cases.

## Introduction

Autoimmune limbic encephalitis is a rare condition characterized by inflammation targeting primarily the limbic structures of the brain, especially the hippocampus and amygdala. It results from an abnormal immune response directed against the central nervous system, leading to an autoimmune attack on neuronal antigens.

Clinically, autoimmune limbic encephalitis typically manifests with diverse symptoms, including cognitive dysfunction, short-term memory loss, confusion, seizures, psychiatric symptoms, or mood disturbances [1,2].

The diagnosis is based on four essential criteria: subacute onset of symptoms progressing within three months; brain abnormalities visible on MRI, particularly in the medial temporal lobes; at least one of the following: cerebrospinal fluid pleocytosis or epileptic activity on electroencephalogram (EEG); and reasonable exclusion of other potential causes [2,3].

## Case presentation

**Figure 1:**
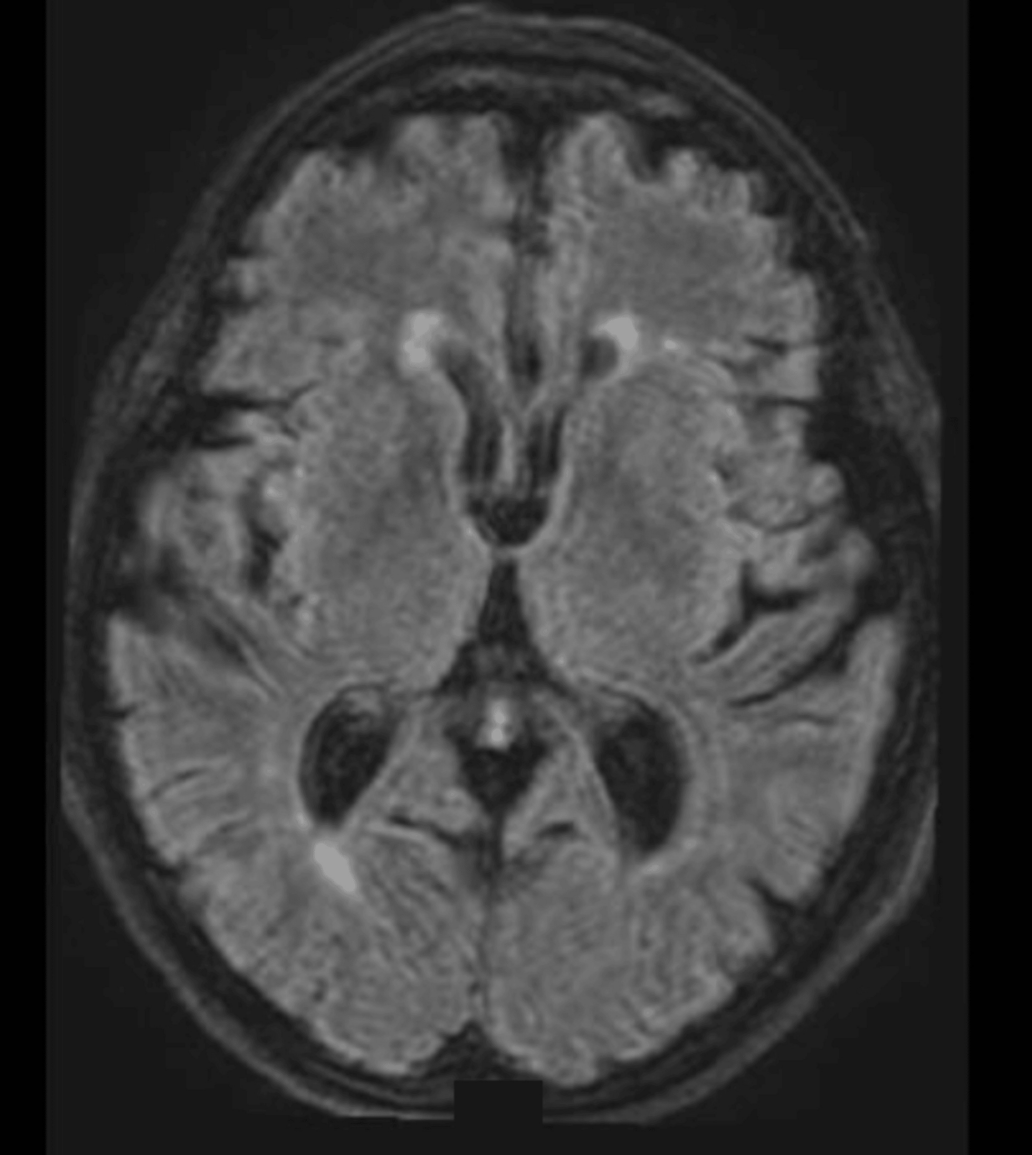
Brain magnetic resonance imaging Axial T2-FLAIR MRI sequence of the brain showing no focal abnormalities, structural lesions, or signal changes that could account for the patient's epileptic seizures. FLAIR: Fluid-attenuated inversion recovery.

We report the case of a 76-year-old female who was initially admitted to the emergency department following a first-time generalized seizure. Recent onset of depression and cognitive impairment was also noted. Initial evaluation included brain MRI (Figure 1)​​​​, EEG, lumbar puncture, and comprehensive tests to rule out infections and vascular, toxic, or metabolic pathologies (Table 1). None of these tests revealed significant abnormalities.

**Table 1:** CSF table results CSF: Cerebrospinal fluid.

Analysis	CSF	Serum	Interpretation
Total proteins	32.3 mg/dL	60 g/L	Normal
Albumin	17.4 mg/dL	38.6 g/L	Normal
IgG	22.8 mg/L	8.4 g/L	Normal
Glucose	76 mg/dL	-	Normal
Lactate	1.61 mmol/L	-	Normal
Isoelectric focusing of IgG	Type 4: no intrathecal IgG synthesis	Identical oligoclonal bands to those in CSF	No intrathecal inflammation
Bacteriology	No germs detected	-	No bacterial infection
PCR for herpes/VZV/enterovirus	Negative	-	No viral encephalitis

**Figure 2:**
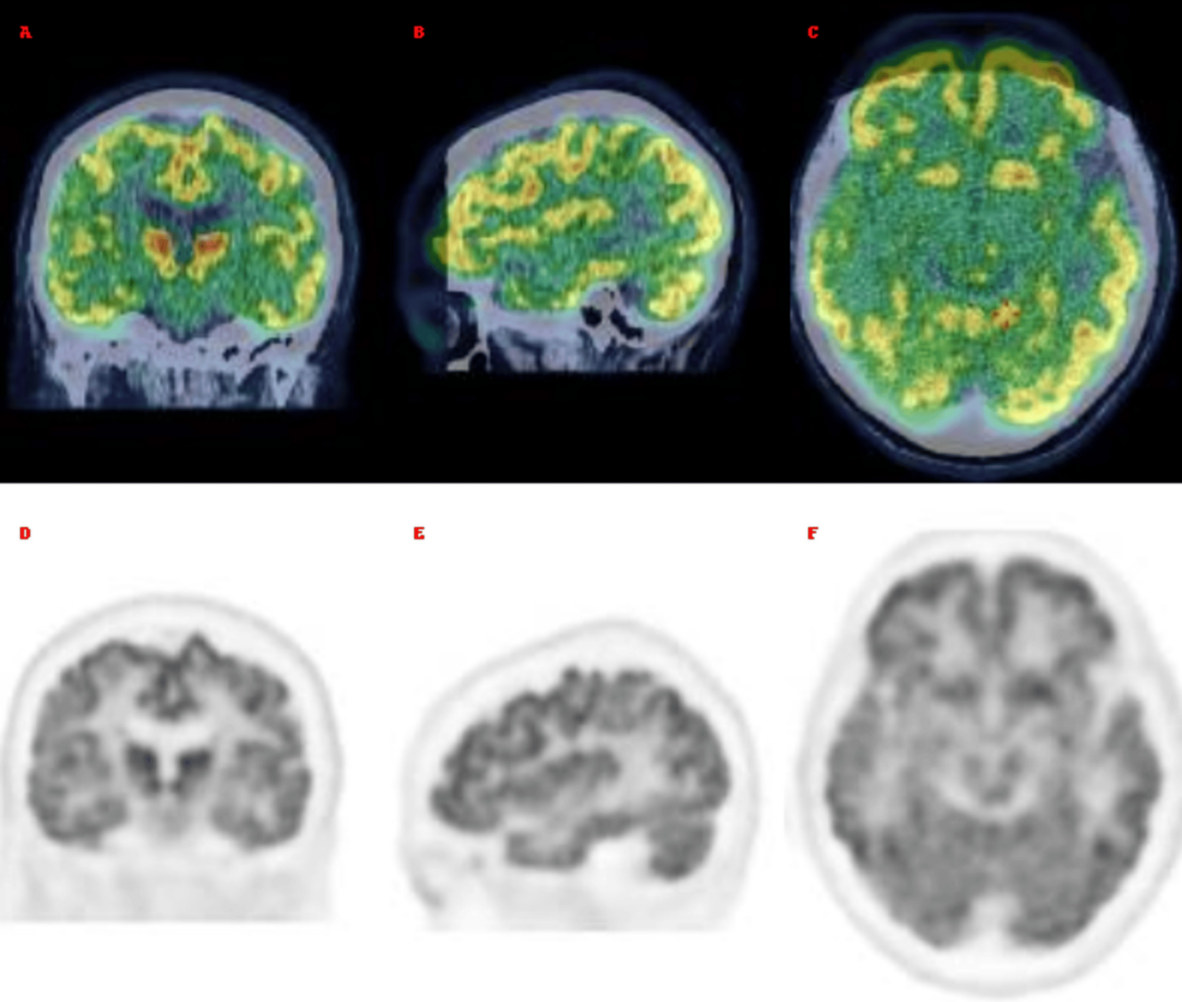
PET of the brain normal (A–C) Fused 18F-FDG PET/CT brain slices in coronal, sagittal, and axial planes, showing normal metabolic distribution. (D–F) Corresponding standalone 18F-FDG PET slices in the same planes, also demonstrating preserved metabolism. 18F-FDG PET/CT: 18F-Fluorodeoxyglucose positron emission tomography/computed tomography.

Under antiepileptic treatment, the patient’s seizures initially improved. However, persistent cognitive symptoms prompted a cerebral 18F-fluorodeoxyglucose positron emission tomography/computed tomography (18F-FDG PET/CT) to be performed under standard resting conditions. Image acquisition was initiated 34 minutes after intravenous injection of the radiotracer (Figure 2), which showed ​​​​​​no evidence of hypermetabolic abnormalities. 

**Figure 3:**
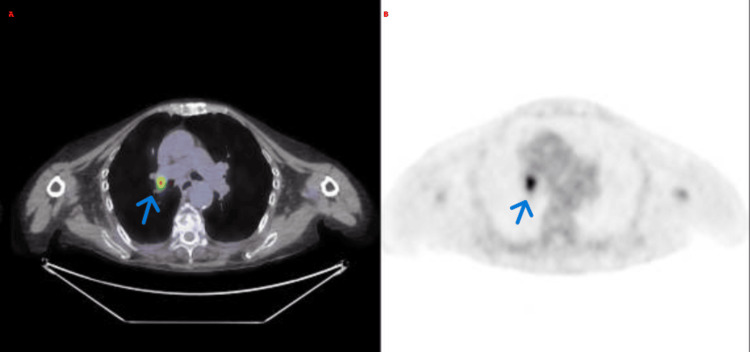
PET/CT of the whole body (A–B) Fused PET/CT and PET-only axial images showing a hypermetabolic lesion in the right supra-hilar pulmonary. PET/CT: Positron emission tomography/computed tomography.

Four weeks later, the patient was readmitted for treatment-resistant seizures. During an evaluation for chronic cough, a CT scan thorax revealed a right para-hilar lung mass. As shown in Figure 3, a whole-body 18F-FDG PET/CT revealed marked hypermetabolism in the right supra-hilar pulmonary (SUV_max_: 6.1).

**Figure 4:**
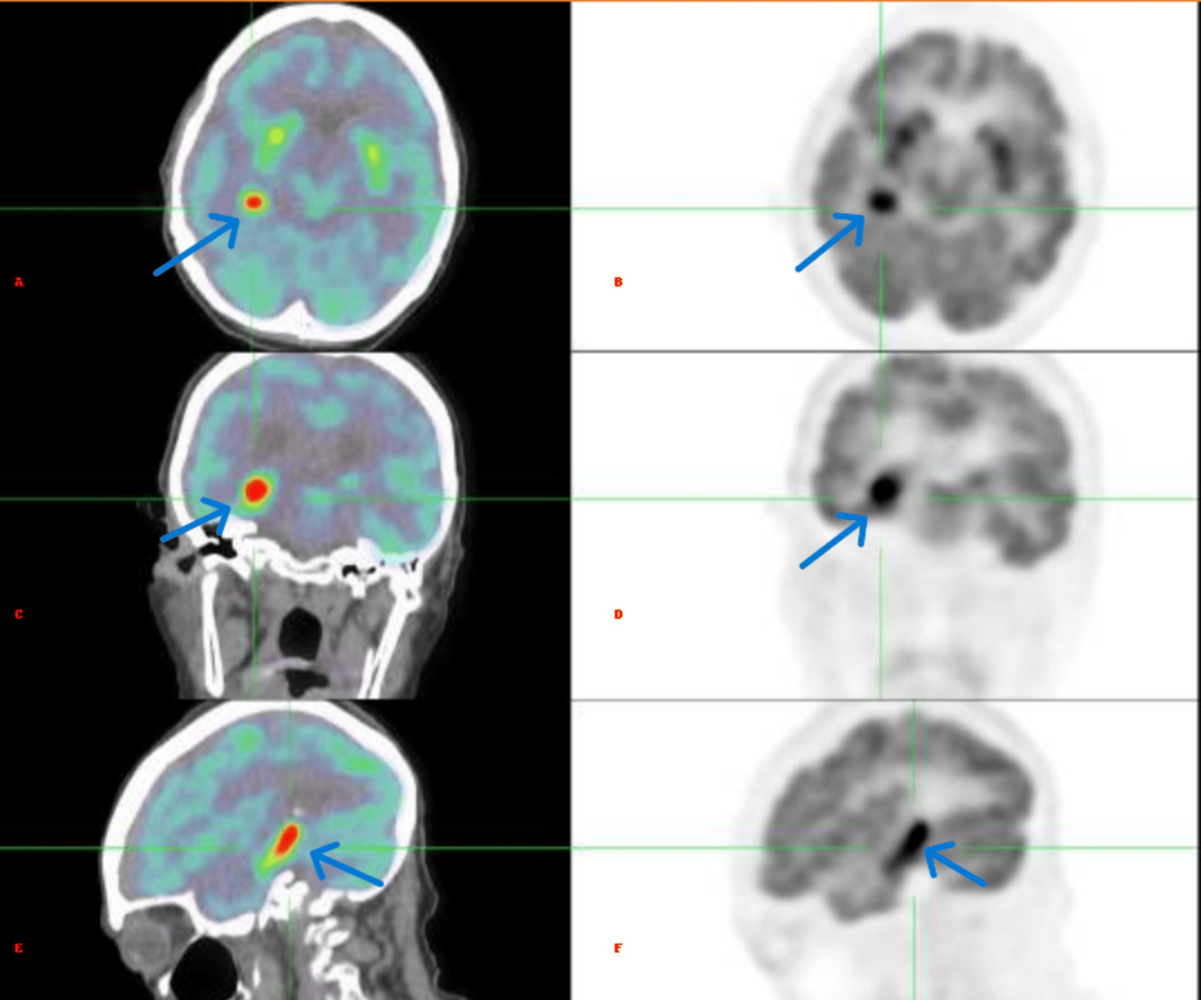
Hypermetabolic temporal lesion (A–C) Fused 18F-FDG PET/CT and (D–F) corresponding PET-only slices in axial, coronal, and sagittal planes, showing a focal hypermetabolic lesion in the right mesial temporal lobe. 18F-FDG PET/CT: 18F-Fluorodeoxyglucose positron emission tomography/computed tomography.

In addition, as shown in Figure 4​​​​​​, a hypermetabolic lesion was observed in the right medial temporal region of the brain, with no corresponding lesion on the CT scan. The administered activity was 2.8 mCi, the patient's blood glucose level was 80 mg/dL, and image acquisition began 59 minutes after the radiotracer injection.

The diagnosis of small-cell lung cancer was established through an endobronchial ultrasound-guided (EBUS) fine-needle aspiration (FNA) of the right hilar pulmonary lesion. Ultimately, the analysis of the CSF sample collected during the initial lumbar puncture, performed four weeks earlier, tested positive for anti-GABAb (anti-gamma-aminobutyric acid type B) antibodies, confirming the diagnosis of paraneoplastic limbic encephalitis.

Clinical improvement was noted following the treatment with Solu-Medrol, leading to significant symptom relief and stabilization of the patient’s condition. In addition to the corticotherapy, the patient was treated with combined radiochemotherapy following the diagnosis of small-cell lung cancer. A follow-up MRI two months later showed no abnormalities, and a subsequent 18F-FDG PET/CT conducted several months later demonstrated complete resolution of the hypermetabolic lesion in the right medial temporal region and also demonstrated complete resolution of the previously hypermetabolic right hilar mass. The patient has not experienced any further seizures to date.

## Discussion

**Figure 5:**
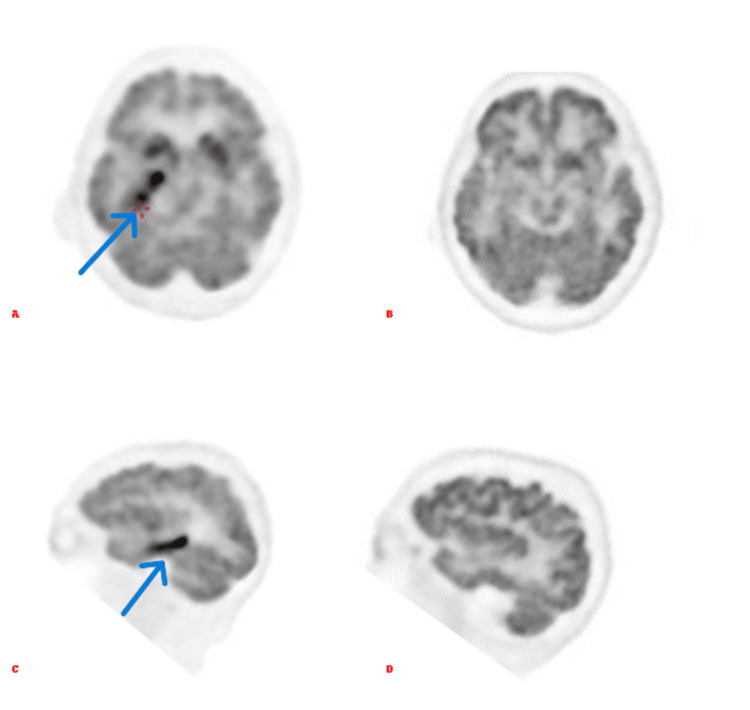
Comparison of FDG brain Comparison of brain 18F-FDG PET images performed at two different time points. (A) Axial view and (C) sagittal view obtained on January 19, 2024, showing the emergence of a focal hypermetabolic lesion in the right mesial temporal region. In contrast, (B) axial and (D) sagittal images from a previous PET scan performed one month earlier demonstrated normal metabolic activity without abnormalities. 18F-FDG PET/CT: 18F-Fluorodeoxyglucose positron emission tomography/computed tomography.

Autoimmune limbic encephalitis is a rare pathology whose diagnosis relies on a combination of clinical presentation, imaging, EEG, and CSF analysis, along with the exclusion of alternative causes [2,3]. These diagnostic tools help identify inflammatory changes in the limbic structures that are characteristic of this condition. However, this case highlights a unique observation: The emergence of a unilateral hypermetabolic lesion in the right medial temporal region on PET/CT imaging was detected (Figure 5) one month after an initial scan that showed no metabolic abnormalities. Notably, both the preceding non-contrast CT scan and MRI failed to demonstrate any structural abnormalities in the same region. On non-contrast CT, autoimmune limbic encephalitis may present as hypodensities in the medial temporal lobes, reflecting underlying inflammation or neuronal injury. MRI typically reveals T2/FLAIR hyperintensities in affected areas, which were absent in this case [4].

This finding suggests the possibility of an early-stage inflammatory lesion. In the initial phases of encephalitis, the inflammatory response may be insufficiently developed to appear in imaging studies or CSF analysis [5]. In our case, the initial MRI and lumbar puncture results were normal, supporting the hypothesis of a progressively subtle inflammatory process [6,7]. Moreover, the favorable clinical response to corticosteroid therapy underscores the inflammatory nature of the hypermetabolic lesion [8]. This suggests that the lesion may have resulted from an inflammatory response triggered by a paraneoplastic mechanism, emphasizing the importance of clinical and imaging follow-up to confirm this hypothesis.

This case also raises the potential need for reevaluation of the diagnostic criteria for autoimmune limbic encephalitis. Specifically, the isolated presence of anti-GABAb antibodies, despite initially negative imaging and CSF results, could be considered a contributory factor to diagnosis [9]. The presence of specific antibodies, even in the absence of other clinical or radiological evidence of inflammation, may allow for earlier identification of impending autoimmune limbic encephalitis. This observation could justify early intervention in patients with suggestive symptoms, even when initial ancillary tests are negative.

Further research is warranted to deepen our understanding of the pathophysiological mechanisms linking hypermetabolism to inflammation and to study the temporal kinetics of its development.

## Conclusions

Our case illustrates an atypical and rare variant of autoimmune limbic encephalitis, marked by the appearance of a unilateral right medial temporal region inflammatory lesion on 18F-FDG PET/CT imaging one month after a negative initial scan. This finding suggests a potential early inflammatory phase of the disease. These observations may contribute to a better understanding of the disease’s evolutionary mechanisms and support the inclusion of anti-GABAb antibodies as a supplemental diagnostic criterion, even in cases where conventional tests are initially negative. Additional studies are necessary to validate our hypothesis.
